# Investigation of the Differential Power of Young's Internet Addiction Questionnaire Using the Decision Stump Tree

**DOI:** 10.1155/2022/3930273

**Published:** 2022-10-14

**Authors:** Mohammad Docharkhehsaz, Touraj Hashemi Nosratabad, Mansour Beirami, Mohammad Taghi Sattari

**Affiliations:** ^1^Department of Psychology, Faculty of Education and Psychology, University of Tabriz, Tabriz, Iran; ^2^Department of Water Engineering, Faculty of Agriculture, University of Tabriz, Tabriz 51666, Iran; ^3^Department of Agricultural Engineering, Faculty of Agriculture, Ankara University, Ankara 06110, Turkey

## Abstract

**Background:**

Internet addiction is one of the serious consequences of recent advances in the use of social media. Early detection of Internet addiction is essential because of its harms and is necessary for timely and effective treatment.

**Aim:**

The aim of this study was to use data mining and an artificial intelligence algorithm to estimate the differential power of each question in the Young Internet Addiction Test and build a decision stump model to predict which item in the questionnaire can be representative of the whole questionnaire.

**Methods:**

This is a descriptive study conducted at the University of Tabriz, in which 256 undergraduate students were selected in randomized cluster sampling, and they completed Young's IAT (Internet Addiction Test) questionnaire and some demographic questions. The data were statistically analyzed with SPSS and were divided into two groups, normal and addicted, by using a cut-off point. Also, the data of the subjects was used to model the decision stump tree in WEKA. The clustering item was the normal and addicted specifier.

**Results:**

The study shows that Cronbach's alpha of the IAT is 0.88, which shows good internal integration of subjects that are used to develop the model in WEKA (the Waikato Environment for Knowledge Analysis). Data analysis showed that by using the second question of this questionnaire as the root of the decision stump tree model, it is possible to distinguish between Internet addicts and healthy users with 82% accuracy using this model.

**Conclusion:**

The study shows innovative ways in which decision stump trees and data mining can help to improve methods used in Clinical Psychotherapy and Human Science. Regarding this, the study showed that early detection of Internet addiction would be possible by using the 2^nd^ question of the IAT. Also, early detection can result in cost-effectiveness for the whole healthcare system.

## 1. Introduction

The Internet is a global system of interconnected computers that uses standard protocols to serve billions of users worldwide. It is an interconnected network of millions of local or global, academic, commercial, and government networks powered by wireless, optical, and electronic technologies. The network has a wide range of information resources accessible via web services and e-mails, and most of the historically conventional media such as telephone, music, film, and television have changed with the advent of the Internet.

As a positive source, the Internet can provide an opportunity to treat anxiety and depression by providing the necessary support and education for the potential patient [1–7]. The Internet has many educational, social, and psychological benefits, and many people have started a variety of businesses online and made economic progress. People have access to a list of available jobs [2]. The Internet can help people improve their lifestyles [8]. The Internet allows for the rapid transfer of information; it also helps to maintain relationships. In addition, it has developed a platform in which emotional support, recreation, online games, and learning about other cultures have become possible [9]. Besides the benefits of the Internet, the disadvantages and negative aspects are also noteworthy. Some people overwork on the Internet so much that they cannot control their use time, and their personal and business relationships suffer greatly [10]. On the other hand, the Internet reduces employee efficiency and shortens the time people can spend with their families. The Internet may give people access to false information and cause psychological problems [4, 5, 9, 11–14]. The reduction in the amount of time people spend with those around them leads to an argument of intolerance and difficulty in relationships.

In defining Internet addiction, Young and Case have stated that overuse and mental occupation with little control to stop using, and the extreme need or behaviors towards computer use and Internet access that cause anxiety [15].

In order to measure and evaluate Internet addiction, clinical efforts have been made based on behavioral semiotics, so that in recent years, this type of addiction has also been considered in the DSM classification system. However, in order to quantitatively evaluate this type of addictive behavior, various tools have been developed in recent years to enable therapists and researchers to accurately measure this phenomenon. One of the most valuable tools is the Young Internet Addiction Questionnaire.

In order to measure Internet addiction, Young developed a questionnaire consisting of 20 questions [16]. The cut-off point of this questionnaire to distinguish between normal and abnormal use of the Internet in Iran is 46 for university students [17] and has been reported at 40 in his research [18].

Although the questionnaire is effective for screening clinical and normal people, in recent years, methods of data analysis from measurement tools have evolved significantly and provide a basis for accurate and rapid assessment of individual characteristics or phenomena using artificial intelligence algorithms, one of which is data mining by computers that are based on artificial intelligence algorithms.

The use of data mining methods and machine learning to detect latent relations between parameters has expanded with the increasing power of computer analysis. The use of decision trees, probabilistic space, and probability decision-making is of particular importance in a real-world problem based on artificial intelligence data mining in clinical treatment studies can lead to identifying hidden, but very crucial, insights for psychologists about their clients. However, the use of data and exploratory methods in psychology is less well known [19].

One of the fastest and most efficient methods in the field of data mining is the use of the decision stump tree method. The decision stump tree works very well on benchmark data from standard machine learning data [20]. This method uses only one attribute for separation. In discrete data, the internal node will be a number. For categorical data, the root nodes will contain a series of leaves. In continuous data, the nodes may be slightly more complex.

Decision trees have advantages over other data mining methods. Decision trees have the ability to work with discrete, categorical, and continuous data. Decision trees are easier for humans to understand. There is no need for the distribution function estimation method. The decision trees follow the white box model while the artificial neural network algorithm uses the black box model. On the other hand, the time spent in decision trees for large data volumes is relatively shorter.

Decision trees also have disadvantages; if the number of samples for training is small, the error rate is high, and if the groups overlap, the number of nodes increases and the error may accumulate from one level to another, increasing the total error.

There are different types of decision trees, but it has been shown that the decision stump in most cases is exactly as perfect as the standard decision tree [20].

Despite the simplicity of the decision stump, its accuracy is logarithmic, which makes it precious and high enough compared to standard decision trees. Also, the presence of peer-to-peer traits makes the effect of noise nonsymmetrical [21].

With the spread of the Internet, its use has also become very widespread. Also, it has greatly increased the amount of data available. Discussing and drawing conclusions from raw data, manually and relying on human resources is a very difficult and sometimes impossible task, so the use of computers, as well as algorithms for the fast and relatively accurate conclusion of data is essential. Data mining is a science through which one can access the content and hidden relationship within raw data. Recently, data mining has been the most important method to use big data efficiently, and its importance is increasing [22].

Data mining explores hidden patterns using a combination of data representing explicit general knowledge, complex data analysis skills, and knowledge specific to a particular field of study. These revealed models for forecasting can be used to extract new horizons from the data [23].

There are many different methods for data mining that make some inferences easier. Among these, decision trees are attractive to researchers and users because they resemble the structure of human thinking. Rules and functions related to the subject can also be easily represented with decision trees.

### 1.1. Decision Stump Tree Model

Tree models are based on divide-and-conquer algorithms. The solution to these problems is based on algorithms that divide the problem into small, solvable problems. Categorization is one of the most important features of such algorithms.

In tree models, the overall structure is similar to an abstract tree, which includes roots, nodes, leaves, and branches. Using the c5 decision tree in small data may be problematic, but decision stumps can be effective when data is scarce [24].

Each of these concepts has a specific meaning in its place. A particular type of decision tree is known as the decision stump. In this type of tree, there is only one level. Also, the main node or root is actually the specific question or case to be decided.

Depending on which condition is obtained, the path is followed on one of the branches and we will reach the result, which in our case is the correct classification of Internet addiction. Decision stump trees are widely used in real-time computer systems to speed up decision-making where time is short and the need for a high-speed decision is considered. Applications include the use of these types of trees in image processing to identify individuals in security systems [25], as well as the detection of cyber-attacks [26].

The use of a single-level tree algorithm has been shown to work very well on naturally collected data. However, the existence of any structures for machine learning on the data, such as data manipulated for easy problem-solving purposes or computer-structured data, can make the algorithm very error-prone [20].

Decision trees have been used for a variety of purposes. In the fields of water engineering [22], diagnosis and classification of electroencephalogram data [27], speech recognition [28], predicting the conversion of cognitive problems to 'Alzheimer's [29], and various other subjects, similar methods have been used. However, this method has been less used in the field of assessment of addiction disorders, so the purpose of this study is to use the decision stump tree to evaluate Internet addiction based on the scores of the Young Internet Addiction Questionnaire and shorten it to apply Internet addiction prediction.

We aimed to design a prediction model for a decision tree that can aid in identifying the most important attribute of Young's IAT questionnaire, which can predict the classification of Internet addiction.

## 2. Methods and Materials

### 2.1. Study Design

The University of Tabriz was founded in 1947 in the city of Tabriz, Iran. This university is one of the national universities of Iran and is one of the oldest universities there. Also, nearly 24,000 students were studying, of which 13200 students were undergraduates [30].

The type of study was descriptive, and standard questionnaires with demographic sections were used. The statistical population was the population of Tabriz University undergraduate students. The subjects were selected by cluster sampling. The selection criteria were to be an undergraduate student at the University of Tabriz and be in the random cluster. The partially answered surveys were left out.

The number of samples selected for this study was 256 people. Questionnaires were distributed in the university classrooms. Each person expressed his/her written consent to participate in the research by filling in the relevant section of the questionnaire. They completed the questionnaire, and the process of gathering data by questionnaire took a week. The conditions for distribution, explanation, and completion of the questionnaire were the same for all samples.

### 2.2. Tools

The questionnaires that were used included the demographic information section and the Young Internet Addiction Questionnaire. The Young Internet Addiction Questionnaire is a questionnaire consisting of 20 questions in which each question has 6 spectral answers, and each answer shows the repetition of the measured content of that question. Answers include “Not Applicable,” “Rarely,” “Occasionally,” “Frequently,” “Often,” and “Always,” and each was assigned an incremental score, respectively.

Internet addiction scores are based on the sum of the scores of each question.  Table 1 provides descriptive statistics for these scores in the selected sample.

This questionnaire has been analyzed in different countries and has gained acceptable validation criteria. Cronbach's alpha of the Internet Addiction Questionnaire in this study was calculated to be 0.88, which indicates the good internal validity of this questionnaire in the statistical population. Based on the research conducted on his questionnaire, Young proposed Table 1 to determine the severity of Internet addiction.

Other research has been carried out by different researchers in different countries to determine the harmfulness of Internet addiction. The cut-off point of 40 in international studies [18] and the cut-off point of 46 in domestic studies [17] have been mentioned for this purpose. A cut-off point is a point above which determines the amount of harmful Internet usage, which can be interpreted as an addiction.

Young points out that a high score on each question can provide good information on the problems associated with Internet addiction.

The decision tree extracted in this study was created using WEKA software version 3.8. WEKA is a tool for data analysis, which has the ability to process data, categorize, chart, and test results [31].

### 2.3. Method

For this purpose, the data extracted from the questionnaires were entered into Excel after initial calculations and then converted to a readable format by WEKA. The data used in this study included questions (20 questions) and answers given by the subjects, along with the classification of Internet addicts as healthy from the cut-off point of 46.

Table 2includes the summary of steps and the calculation methods used in WEKA.

## 3. Results

Preliminary calculations of the addiction score obtained from the Young Questionnaire are given in Table 3.

Questions 1 to 20 were named e1 to e20, respectively. WEKA modeled the data, and the question number 2, e2, was selected as the root of the decision stump tree.

This question states:

“*How often do you neglect household chores to spend more time online*?”(Appendix 1).

Based on this and the rules produced, it is determined that if the subject answers the second question and gets a score higher than 3.5 (in this case, it means “Often” and “Always”), then he will be in the group of Internet addicts. Whereas, if he chooses an answer with a lower score, then the subject will be classified as having healthy use of the Internet (answers: “Not Applicable,” “Rarely,” “Occasionally,” “Frequently”).

**Figure 1:**
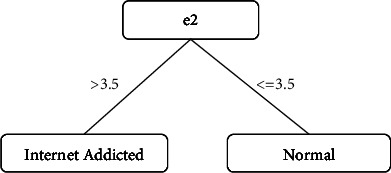
Calculated root decision tree. Decision tree classification: *e*2 ≤ 3.5: normal. *e*2 > 3.5: Internet addict. If *e*2 is not available: normal.

The decision tree created is shown in Figure 1.

The resulting decision tree, using 10 replications, was able to categorize 208 of the 256 available samples based on a correctly calculated model. (81.3% accuracy of classification) and only 48 items were incorrectly classified.  Table 4 shows the confusion matrix.

Table 5 shows the complete specifications for the accuracy of the decision stump tree prediction model with E2 as its root.

**Figure 2:**
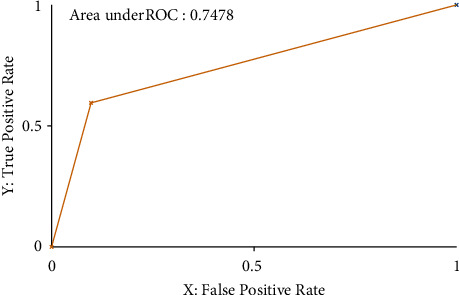
Threshold curve and ROC–class Internet addicted.

**Figure 3:**
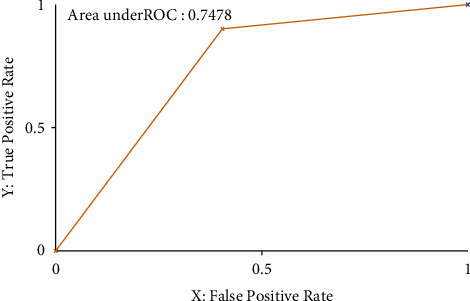
Threshold curve and ROC–class normal.

Also, the ROC (receiver operating characteristic) value and threshold curve plot for both normal and Internet-addicted are shown in Figures 2 and 3.

## 4. Discussion

The aim of the study was to analyses the IAT using data mining and the machine learning algorithm decision stump tree. So, the consistency of the questioner in the sample population was determined.

The study has a good Cronbach's alpha for the Internet Addiction Questionnaire (0.88). This indicates the good internal validity of this questionnaire in the statistical population. So, the items in the questionnaire accurately test the things that the questionnaire is supposed to. The statistical and descriptive information of the study was shown in Table 2. The training and modeling of the data are performed in WEKA, and it resulted in Figure 1.

The results show that entire questionnaire items can be reduced to one question, which is question number 2. The accuracy of this model in accurately classifying Internet addiction is 0.81 (Table 5). The TP (true positive) rate in Table 5 shows a true positive value of 0.595 for the Internet addicted group, which shows 59.5 percent of the Internet addicts' population is classified as Internet addicted by the trained decision stump tree model. Also, the TP Rate of 0.901 shows 90.1 percent of normal people are truly classified by this model.

The results showed that this model can accurately predict. The accuracy of the results is = 80.6% and the model has a representational power of 81.3%.

In machine learning systems, the most important parameter is ROC. Roc can take values between −1 and +1, and the closer to 1, the more accurate the model is. The area below the diagram is an important parameter that was calculated at 0.748 in this study, which is comparatively acceptable [32–34]. Also, a high MMC (Moving Morphable Component) of 0.525 indicates that the predicted model, which is created, was reliable.

## 5. Conclusion

Since the method used in this study was quite new to this field, the study has shown that it can be used for modeling and identifying the main factors of a questionnaire, which are based on machine learning and new computational methods.

Using the tree decision tree method, appropriate predictive models can be found that are also understandable to humans. The important point in this computational method is its simplicity and ease of using the results, along with the identification of the hidden relationships between the data. This study showed how the root decision tree can be used to create a high-precision model for IAT.

The IAT questionnaire is a validated test for detecting and identifying Internet addiction that has been used in many studies and in many clinical institutes as a method for assessing Internet addiction. The need for studying its subset factors, which are in 20 questions, and modeling subset factors as classifying items was performed in this study. Using new machine learning and data mining algorithms in the psychological assessment field is an innovative way, which makes this current study novel. So, the methods which are used in this research can serve as a guideline for new computational psychology studies.

## 6. Implications and Limits

The study shows innovative ways in which decision stump trees and data mining can help to improve methods used in Clinical Psychotherapy and Human Science. Regarding this, the study showed that early detection of Internet addiction would be available by the 2^nd^ question of the IAT. Also, early detection can result in the cost-effectiveness of the whole health system.

The findings of this study can be used as a quick way to identify and correlate major problems in mental disorders. Thus, in the model obtained from this study, it was shown that “not doing daily home activities excessively predicts harmful Internet addiction.” Therefore, by building appropriate cognitive therapy protocols, patients can minimize the harms of Internet addiction by engaging in household chores and solving basic problems in this area.

The created model was able to reduce the number of questions in the Young Questionnaire online to a basic question with high accuracy. However, according to the sample selection method, which is not completely random, and the target population, which is limited, the results should be used carefully and excessive generalization should be avoided.

For further work, these algorithms can be used on other diagnostic questionnaires and the range of questions can be reduced. For example, it can be used for questionnaires about suicidal behavior, anxiety, or depression.

Early detection of Internet addiction is essential because of its harms, and it is necessary for timely and effective treatment. The study can help healthcare institutions to assess and identify the problems in a quick manner, as psychologists in educational institutes or government departments can easily classify Internet addicts.

## Figures and Tables

**Table 1:** IAT score and Internet addiction.

	Score
Normal range	0–30
Mild	31–49
Moderate	50–79
Severe	80–100

**Table 2:** Summary of steps.

Action taken:
(1) Sample selection was done by cluster random sampling.
(2) Questionnaires were distributed and results were obtained.
(3) The data was entered into the computer in Excel software.
(4) Internet addiction score calculations were performed in Excel.
(5) Individuals were labeled according to the score obtained into two groups: healthy and Internet addicted.
(6) The data prepared for the accepted format as WEKA.
(7) The decision stump algorithm was applied to the data according to using train set option in WEKA (test on the same set that classifier is trained on) for training and testing.
(8) The results were extracted.

**Table 3:** Descriptive statistics of Internet addiction scores.

	*N*	Minimum	Maximum	Mean	Std. deviation
Internet addicted	74	46.00	82.00	54.8649	8.14816
Normal	182	4.00	45.00	31.4286	9.10970
Total	256	4.00	82.00	38.2031	13.8286

**Table 4:** Confusion matrix.

Classified as=>	*a*	*b*
*a* = internet addicted	44	30
*b* = normal	18	164

**Table 5:** Detailed accuracy by class.

	TP rate	FP rate	Precision	Recall	*F*-measure	MMC	ROC area	RPC area	Class
	0.595	0.099	0.710	0.595	0.647	0.525	0.748	0.539	Internet addicted
	0.901	0.405	0.845	0.901	0.872	0.525	0.748	0.832	Normal
Weighted avg	0.813	0.317	0.806	0.813	0.807	0.525	0.748	0.747	

## Data Availability

The raw data used in this study will be made available by the authors to other qualified researchers.

## Additional Points

*Limitations*. These new computational models should be localized by the researchers since the type of this study is not quite random and over generalization should be avoided.

## Supplementary Materials

Supplementary Materials Appendix 1: Young Internet Addiction Test.
