# Supplementary material for: Investigation of the Differential Power of Young's Internet Addiction Questionnaire Using the Decision Stump Tree
Source: Comput Intell Neurosci. 2022 Oct 14;2022:3930273. doi: 10.1155/2022/3930273 (PMC9586745; doi:10.1155/2022/3930273)
Supplement: Supplementary Materials — Appendix 1: Young Internet Addiction Test. [file 3930273.f1.pdf]

INTERNET ADDICTION TEST

Name____________________________
Male______Female ________
Age_______Years Online _______ Do you use the Internet for work?_______Yes_______No

This questionnaire consists of 20 statements. After reading each statement carefully, based upon the 5-point Likert scale, please select the response (0, 1, 2, 3, 4 or 5) which best describes you. If two choices seem to apply equally well, circle the choice that best represents how you are most of the time during the past month. Be sure to read all the statements carefully before making your choice. The statements refer to offline situations or actions unless otherwise specified.

0 = Not Applicable
1 = Rarely
2 = Occasionally
3 = Frequently
4 = Often
5 = Always

1. ___How often do you find that you stay online longer than you intended?
2. ___How often do you neglect household chores to spend more time online?
3. ___How often do you prefer the excitement of the Internet to intimacy with your partner?
4. ___How often do you form new relationships with fellow online users?
5. ___How often do others in your life complain to you about the amount of time you spend online?
6. ___How often do your grades or school work suffer because of the amount of time you spend online?
7. ___How often do you check your email before something else that you need to do?
8. ___How often does your job performance or productivity suffer because of the Internet?
9. ___How often do you become defensive or secretive when anyone asks you what you do online?
10. ___How often do you block out disturbing thoughts about your life with soothing thoughts of the Internet?
11. ___How often do you find yourself anticipating when you will go online again?
12. ___How often do you fear that life without the Internet would be boring, empty, and joyless?
13. ___How often do you snap, yell, or act annoyed if someone bothers you while you are online?
14. ___How often do you lose sleep due to being online?
15. ___How often do you feel preoccupied with the Internet when off-line, or fantasize about being online?
16. ___How often do you find yourself saying "just a few more minutes" when online?
17. ___How often do you try to cut down the amount of time you spend online and fail?
18. ___How often do you try to hide how long you've been online?
19. ___How often do you choose to spend more time online over going out with others?
20. ___How often do you feel depressed, moody, or nervous when you are off-line, which goes away once you are back online?

COPYRIGHT © by Dr. Kimberly S. Young
All rights reserved. Center for Internet Addiction Recovery, P.O. Box 632, Bradford, PA 16701
